# T-SPOT.TB Reactivity in Southern African Children With and Without *in Utero* Human Immunodeficiency Virus Exposure

**DOI:** 10.1093/cid/ciad356

**Published:** 2023-06-09

**Authors:** Saori C Iwase, Paul T Edlefsen, Lynnette Bhebhe, Kesego Motsumi, Sikhulile Moyo, Anna-Ursula Happel, Danica Shao, Nicholas Mmasa, Sara Schenkel, Melanie A Gasper, Melanie Dubois, Megan A Files, Chetan Seshadri, Fergal Duffy, John Aitchison, Mihai G Netea, Jennifer Jao, Donald W Cameron, Clive M Gray, Heather B Jaspan, Kathleen M Powis

**Affiliations:** Division of Immunology, Department of Pathology, University of Cape Town, Cape Town, South Africa; Institute of Infectious Disease and Molecular Medicine, University of Cape Town, Cape Town, South Africa; Vaccine and Infectious Disease Division, Statistical Center for HIV/AIDS Research and Prevention, Fred Hutchinson Cancer Research Center, Seattle, Washington, USA; Botswana Harvard AIDS Institute Partnership, Gaborone, Botswana; Botswana Harvard AIDS Institute Partnership, Gaborone, Botswana; Botswana Harvard AIDS Institute Partnership, Gaborone, Botswana; Department of Immunology and Infectious Diseases, Harvard T.H. Chan School of Public Health, Boston, Massachusetts, USA; Division of Immunology, Department of Pathology, University of Cape Town, Cape Town, South Africa; Institute of Infectious Disease and Molecular Medicine, University of Cape Town, Cape Town, South Africa; Vaccine and Infectious Disease Division, Statistical Center for HIV/AIDS Research and Prevention, Fred Hutchinson Cancer Research Center, Seattle, Washington, USA; Surgical Department, County Durham and Darlington NHS Trust, Darlington Memorial Hospital, Darlington, United Kingdom; Division of Pediatric Global Health, Massachusetts General Hospital, Boston, Massachusetts, USA; Center for Global Infectious Disease Research, Seattle Children’s Research Institute, Seattle, Washington, USA; Division of Pediatric Global Health, Massachusetts General Hospital, Boston, Massachusetts, USA; Division of Infectious Diseases, Department of Pediatrics, Boston Children's Hospital, Boston, Massachusetts, USA; Department of Medicine, University of Washington School of Medicine, Seattle, Washington, USA; Department of Medicine, University of Washington School of Medicine, Seattle, Washington, USA; Center for Global Infectious Disease Research, Seattle Children’s Research Institute, Seattle, Washington, USA; Center for Global Infectious Disease Research, Seattle Children’s Research Institute, Seattle, Washington, USA; Department of Internal Medicine and Radboud Center for Infectious Diseases, Radboud University Medical Center, Nijmegen, The Netherlands; Department of Immunology and Metabolism, Life & Medical Sciences Institute, University of Bonn, Bonn, Germany; Botswana Harvard AIDS Institute Partnership, Gaborone, Botswana; Department of Pediatrics, Department of Medicine, Northwestern University Feinberg School of Medicine, Chicago, Illinois, USA; Divisions of Infectious Diseases and Respirology, University of Ottawa at the Ottawa Hospital, Ottawa, Canada; Division of Immunology, Department of Pathology, University of Cape Town, Cape Town, South Africa; Institute of Infectious Disease and Molecular Medicine, University of Cape Town, Cape Town, South Africa; Division of Molecular Biology and Human Genetics, Biomedical Research Institute, Stellenbosch University, Cape Town, South Africa; Division of Immunology, Department of Pathology, University of Cape Town, Cape Town, South Africa; Institute of Infectious Disease and Molecular Medicine, University of Cape Town, Cape Town, South Africa; Center for Global Infectious Disease Research, Seattle Children’s Research Institute, Seattle, Washington, USA; Botswana Harvard AIDS Institute Partnership, Gaborone, Botswana; Department of Immunology and Infectious Diseases, Harvard T.H. Chan School of Public Health, Boston, Massachusetts, USA; Departments of Internal Medicine and Pediatrics, Massachusetts General Hospital, Boston, Massachusetts, USA

**Keywords:** TB infection, Southern Africa, infants, HIV exposure, T-SPOT.TB

## Abstract

Infants who are human immunodeficiency virus (HIV)-exposed uninfected (iHEU) experience higher risk of infectious morbidity than infants HIV-unexposed uninfected (iHUU). We compared tuberculosis (TB) infection prevalence in 418 Bacillus Calmette-Guérin vaccinated sub-Saharan African iHEU and iHUU aged 9–18 months using T-SPOT.TB. Prevalence of TB infection was low and did not differ by HIV exposure status.

Children under 5 years of age experience high risk of progression to tuberculosis (TB) disease if untreated [1]. In sub-Saharan Africa, about 25% of persons of childbearing potential are diagnosed with human immunodeficiency virus (HIV) [2]. Successful antiretroviral treatment (ART) scale-up for pregnant persons with HIV has resulted in an increasing number of infants born HIV-exposed uninfected (iHEU).The iHEU experience greater risk of infectious morbidity than infants born HIV-unexposed uninfected (iHUU) [3].

Bacillus Calmette-Guérin (BCG) vaccine prevents severe TB disease. BCG vaccination induces T-cell interferon-gamma (IFN-γ) production, an important component of protection against TB [4]. We previously found a significantly lower proportion of BCG-specific IFN-γ producing CD4^+^ T cells among iHEU, suggesting that iHEU may not achieve equivalent BCG immune protection compared to iHUU [5]. Therefore, we investigated TB infection prevalence by HIV exposure status among BCG-vaccinated infants in Botswana and South Africa (SA), 2 high burden HIV and TB settings.

## METHODS

### Study Design

The study was nested within 2 prospective observational cohort studies enrolling pregnant women with and without HIV and their infants. The Tshilo Dikotla study and the Innate Factors Associated with Nursing Transmission (InFANT) study recruited participants from government antenatal clinics in Botswana and SA between 2013 and 2020 [6, 7]. Infants with severe birth complications, or born to mothers with active TB or TB symptoms were excluded. All infants received BCG vaccination within 72 hours of birth. Participants were followed over 36 months in Botswana and 12 months in SA. Peripheral blood mononuclear cells were collected at 9–12 and 18 months in Botswana, at 9 and 12 months in SA, and stored in liquid nitrogen.

### Ethics

This study was approved by the Health Research Development Committee in Botswana, Massachusetts General Hospital's Institutional Review Board, and University of Cape Town's Human Research Ethics Committee. Women provided written informed consent for their participation and that of their infant.

### T-SPOT.TB Assay

T-SPOT.TB assays (Oxford Immunotec) were performed and interpreted according to manufacturer's instructions. Samples below the recommended cell number were normalized as previously described [8]. For invalid or borderline results, re-testing was performed using an aliquot collected at the same visit or a follow-up visit. If the re-tested result was valid, the valid result was assigned to the initial visit. Infants testing T-SPOT.TB positive were referred to government clinics. We defined “TB infection” as T-SPOT.TB positive without TB disease symptoms at the time of specimen draw.

### Sensitivity Analysis

Due to timing variation of testing between sites, we simulated results as if all testing was performed at 12 months. For SA infants, we assumed a positive test at month 9 would have a negative at month 12 with probability R (reversion), and infants with a negative or invalid result at month 9 would have a positive at month 12 with probability C (conversion). These probabilities were applied to 12- to 18-months results in the Botswana cohort. We assumed a baseline P (prevalence) at 12 months to calculate the probability of a positive (or negative/invalid) test at 18 months having been negative (or positive) at month 12. For each combination of R, C, and P, ranging from 0% to 20% based on published studies [8, 9], we randomly generated 5000 data sets and performed Fisher exact tests on the pooled month 12 data, comparing the proportion of positive results by infant HIV exposure status.

### Statistical Analysis

Data analysis was performed using R (version 4.0.4). Normally distributed continuous variables were compared by *t* test using means with standard deviations. Continuous variables with skewed distributions were compared by Wilcoxon rank-sum test using medians with interquartile ranges. Categorical variables were compared by χ^2^ test. TB infection proportions were compared by infant HIV exposure status using Fisher exact test.

### Power Calculations

Previous sub-Saharan data reported a prevalence of TB infection of 10.9% (95% confidence interval [CI], 6.1%–17.7%) in 6-month-old iHEU [8]. Thus, we expected that at least 18% of iHEU would test positive by 12 months. Given our study's sample size (125 iHUU and 293 iHEU), we had at least 80% power to detect a 57.5% difference in TB infection at 12 months, assuming a prevalence of ≤ 7.65% among iHUU.

## RESULTS

### Cohort Characteristics

The study included 418 mother-infant pairs, of which 293 were iHEU (Supplementary Table 1). The proportion of iHEU was higher in Botswana compared to SA. Infant sex and gestational age at birth were similar between HIV exposure groups. Women with HIV were older and had higher gravidity than women without HIV. Among women with HIV, 63.0% were on ART at conception with median CD4 count of 463 cells/mm^3^ at enrollment. Fifteen (3.6%) infants had household TB exposure cases (n = 5 in Botswana; n = 10 in SA), including 6 mothers of infants in SA. Household exposure did not differ by HIV exposure status.

### Prevalence of TB Infection

The 418 infants’ results comprised 14 (3.3%) T-SPOT.TB positive, 1 (0.24%) borderline, 15 (3.6%) invalid, and 388 (92.8%) negative (Table 1). T-SPOT.TB reactivity did not differ by infant HIV exposure status (Table 1) or by study site (Supplementary Table 2). No infants who tested positive and were referred for clinical evaluation were diagnosed with TB disease. Two reversions (0.48%) occurred among Botswana iHEU, 1 of which had a household TB contact (Supplementary Table 1). Although spot-forming cells (SFCs) for TB antigens did not differ pre-reversion, SFCs for phytohemagglutinin (positive control) were significantly lower than other positive cases when they reverted (median 165 vs 724, *P* = .003).

**Table 1.** T-SPOT.TB Reactivity by HIV Exposure Status

		iHUU	iHEU
		N = 125	N = 293
Study site, n (%)	Botswana	33 (26.4)	135 (46.1)
	SA	92 (73.6)	158 (53.9)
Testing time point, n (%)	Month 9	44 (35.2)	117 (39.9)
	Month 12	48 (38.4)	46 (15.7)
	Month 18	33 (26.4)	130 (44.4)
T-SPOT.TB result, n (%)	Positive^a^	4 (3.2)	10 (3.4)
	Negative^b^	115 (92.0)	273 (93.2)
	Borderline^c^	0 (0.0)	1 (0.3)
	Invalid^d,e^	6 (4.8)	9 (3.1)

Abbreviations: HIV, human immunodeficiency virus; iHUU, HIV-unexposed uninfected infants; iHEU, HIV-exposed uninfected infants; PHA, phytohemagglutinin; SA, South Africa; SFCs, spot-forming cells; TB, tuberculosis.

Positive if there were ≥ 8 SFCs above Nil control for at least 1 of TB antigens.

Negative if a test did not fall into any of the interpretations.

Borderline if the difference to Nil control was between 5–7 SFCs for at least 1 of TB antigens.

Invalid if there were PHA < 20 SFCs or Nil control > 10 SFCs.

Reason of invalid: contamination of kits or assay (n = 2); PHA < 20 SFCs (n = 7) and Nil control > 10 SFCs (n = 6).

We conducted a sensitivity analysis to impute T-SPOT.TB results at 12 months of age across study sites, considering potential conversion and reversion rates over time (Supplementary Table 3). No combination of assumptions gave a statistically significant difference between HIV-exposure groups in T-SPOT.TB positivity more than 5% of the time.

## DISCUSSION

In our Southern African cohort, we found a low overall risk of TB infection among infants BCG-vaccinated at birth (3.3%), without variation by HIV exposure status. The lack of difference is important, as iHEU have been reported to be at high risk of infectious morbidity [3]. Although testing was performed between 9 and 18 months of life, with some infants having a longer window of risk for TB exposure, the sensitivity analysis suggests that the prevalence of T-SPOT.TB positivity was robust to conversion and reversion between the observed time points. Literature investigating TB infection in iHEU using interferon-gamma release assays (IGRAs) like the QuantiFERON-TB or T-SPOT.TB is limited [10], and studies including iHUU as a comparison group are scarce. To our knowledge, this is the largest study comparing TB infection prevalence between Southern African iHEU and iHUU using T-SPOT.TB.

The prevalence of TB infection was lower among infants in this study compared to other studies using IGRA-based approaches [9]. Differences in cohort characteristics likely account for lower IGRA positivity in our study. We excluded mothers with active TB disease. Furthermore, the previously published SA study assessed TB infection in children with a mean age of 3.5 years [9], evaluating a longer exposure window. It was also conducted during a period when SA recorded its highest TB incidence in the last 2 decades [11]. Thus, household TB contact was more common than our study (13.2% vs 3.6%).

We found no difference in T-SPOT.TB reactivity by HIV exposure status. This differs from a Ugandan study where children HEU up to 5 years of age had higher IGRA positivity prevalence than children HUU [10]. Differences in maternal inclusion criteria and longer follow-up period likely explain the higher prevalence reported in Ugandan children who were HEU.

The strengths of this study include a large sample size, with cohorts recruited in neighboring countries, both with high HIV and TB burden, using a common protocol. Pooling of data increased study power. Although the timing of testing varied between sites, we employed a sensitivity analysis to assess for robustness of findings. Given the lower than anticipated T-SPOT.TB positivity prevalence, we did not have sufficient power to conclusively evaluate the association between HIV exposure and TB infection. Because the prevalence of a T-SPOT.TB reactivity in iHEU was 3.4% in our cohort, prevalence among iHUU would had to have been ≤0.175%, a 94.9% reduction, to detect a significant difference. Adequately powered studies would be needed to definitively exclude a higher risk of TB infection in iHEU.

We employed IGRA-based testing, similar to previous studies [9, 10]. Infant T cells have lower IFN-γ producing capacity than adult T cells [12], and perinatal HIV exposure has been associated with altered immunity [13]. Thus, it is unclear whether iHEU and iHUU have similar T-SPOT.TB reactivity. Furthermore, IGRA testing is not recommended for children under 2 years of age, but tuberculin skin testing can result in false positive tests in BCG-vaccinated individuals. Assays targeting non-IFN-γ markers have been proposed as alternatives in BCG-vaccinated children under 2 years of age [14] and may be beneficial to use in future studies in parallel with IGRA testing.

In summary, we showed that the TB infection prevalence among BCG-vaccinated infants from 2 Southern African countries with high HIV and TB prevalence was low and did not vary by fetal HIV exposure status.

## Supplementary Data

Supplementary materials are available at *Clinical Infectious Diseases* online. Consisting of data provided by the authors to benefit the reader, the posted materials are not copyedited and are the sole responsibility of the authors, so questions or comments should be addressed to the corresponding author.

## Supplementary Material

ciad356_Supplementary_Data

## Notes

***Acknowledgments.*** The authors thank the mothers and infants who participated in this study, the research and lab staff at Botswana Harvard AIDS Institute Partnership in Gaborone, Botswana, and the clinic Maternal Obstetric Unit in Cape Town, South Africa.

***Previously presented*.** HIV & Pediatrics 2022, Montreal, Canada, July 2022. Abstract number 38. AIDS 2022, Montreal, Canada, August 2022. Abstract number PEMOB32.

***Financial support.*** This work was supported by the National Institute of Allergies and Infectious Disease (NIAID) (grant number R01AI142670 awarded to K. M. P.). The Tshilo Dikotla study was funded by the National Institute of Diabetes and Digestive and Kidney Diseases (grant number R01DK109881 awarded to J. J.). The InFANT study was supported in part by the Global Health Research Initiative (GHRI), a research funding partnership composed of the Canadian Institutes of Health Research, the Canadian International Development Agency, and the International Development Research Centre (grant number THA-118568 awarded to H. B. J. and C. M G.), as well as the NIAID (grant number R01AI120714-01A1 awarded to H. B. J.) and National Institute of Child Health and Human Development (NICHD) (grant number R21HD083344 awarded to H. B. J. and C. M. G.). M. D. was supported by NIAID (grant number T32AI007433). S. C. I. was funded by Yoshida Scholarship Foundation.
