# Supplementary material for: T-SPOT.TB Reactivity in Southern African Children With and Without in Utero Human Immunodeficiency Virus Exposure
Source: Clin Infect Dis. 2023 Jun 9;77(8):1133–6. doi: 10.1093/cid/ciad356 (PMC10573724; doi:10.1093/cid/ciad356)
Supplement: ciad356_Supplementary_Data [file ciad356_supplementary_data.zip › Supplementary Table 1.docx]

**Supplementary Table 1. Comparison of overall cohort characteristics by HIV exposure status.**

|  |  | **iHUU** | **iHEU** | ***P*** |
| --- | --- | --- | --- | --- |
|  |  | **N = 125** | **N = 293** |  |
| Study site, n (%) | Botswana | 33 (26.4) | 135 (46.1) | <0.001 |
|  | SA | 92 (73.6) | 158 (53.9) |  |
| Household water accessibility, n (%)^b^ |  | 85 (70.8) | 206 (71.0) | 1 |
| **Maternal characteristics** |  |  |  |  |
| Age at delivery, years (mean (SD))^a^ |  | 26.05 (4.60) | 29.67 (5.45) | <0.001 |
| Marital status, n (%)^c^ | Married | 25 (21.0) | 37 (12.8) | 0.038 |
|  | Living together | 15 (12.6) | 65 (22.4) |  |
|  | Separated | 0 (0.0) | 1 (0.3) |  |
|  | Single | 79 (66.4) | 187 (64.5) |  |
| Completed at least secondary education, n (%)^d^ |  | 111 (92.5) | 208 (73.2) | <0.001 |
| Employed, n (%)^a^ |  | 59 (47.2) | 128 (43.7) | 0.41 |
| Number of prior pregnancies (median [IQR])^e^ |  | 1.00 [0.00, 2.00] | 2.00 [1.00, 2.00] | <0.001 |
| On ART at conception, n (%)^f^ |  | - | 184 (63.0) | - |
| CD4 count at enrollment, cells /mm^3^ (median [IQR])^h^ |  | - | 463 [323, 622] | - |
| Proportion with HIV viral suppression (<40 copies/mL), n (%)^i^ |  |  | 177 (93.2) | - |
| Developed active TB during study, n (%) |  | 1 (0.8) | 5 (1.7) | 0.79 |
| **Infant characteristics** |  |  |  |  |
| Male sex, n (%)^j^ |  | 58 (48.7) | 142 (49.0) | 1 |
| Gestational age at birth, weeks (median [IQR])^k^ |  | 39.64 [38.61, 40.57] | 39.29 [38.00, 40.29] | 0.17 |
| Household TB contact, n (%) |  | 3 (2.4) | 12 (4.1) | 0.57 |

Abbreviations: iHUU, HIV-unexposed uninfected infants; iHEU, HIV-exposed uninfected infants; IQR, interquartile range; SD, standard deviation; SA, South Africa; ART, antiretroviral treatment; VL, HIV viral load; TB, Tuberculosis.

^a^Missing data from 8 infants (iHUU, n = 5; iHEU, n = 3).

^b^Missing data from 15 infants (iHUU, n = 6; iHEU, n = 9).

^c^Missing data from 9 infants (iHUU, n = 6; iHEU, n = 3).

^d^Missing data from 14 infants (iHUU, n = 5; iHEU, n = 9).

^e^Missing data from 13 infants (iHUU, n = 7; iHEU, n = 6).

^f^Missing data from 1 iHEU infant.

^g^Missing data from 5 iHEU infants.

^h^Missing data from 19 iHEU infants.

^i^Missing data from 102 iHEU infants.

^j^Missing data from 9 infants (iHUU, n = 6; iHEU, n = 3).

^k^Missing data from 10 infants (iHUU, n = 7; iHEU, n = 3).
