# Supplementary material for: T-SPOT.TB Reactivity in Southern African Children With and Without in Utero Human Immunodeficiency Virus Exposure
Source: Clin Infect Dis. 2023 Jun 9;77(8):1133–6. doi: 10.1093/cid/ciad356 (PMC10573724; doi:10.1093/cid/ciad356)
Supplement: ciad356_Supplementary_Data [file ciad356_supplementary_data.zip › Supplementary Table 2.docx]

**Supplementary Table 2. T-SPOT.TB results by study sites.**

|  |  | **South Africa** | |  | **Botswana** | |
| --- | --- | --- | --- | --- | --- | --- |
|  |  | **iHUU** | **iHEU** |  | **iHUU** | **iHEU** |
|  |  | N = 92 | N = 158 |  | N = 33 | N =135 |
| **Testing time point, n (%)** | Month 9 | 44 (47.8) | 117 (74.1) |  | - | - |
|  | Month 12 | 48 (52.2) | 41 (25.9) |  | 0 (0.0) | 5 (3.7) |
|  | Month 18 | - | - |  | 33 (100.0) | 130 (96.3) |
| **T-SPOT.TB result (%)** | Positive^a^ | 3 (3.3) | 4 (2.5) |  | 1 (3.0) | 6 (4.4) |
|  | Negative^b^ | 85 (92.4) | 146 (92.4) |  | 30 (90.9) | 127 (94.1) |
|  | Borderline^c^ | 0 (0.0) | 0 (0.0) |  | 0 (0.0) | 1 (0.7) |
|  | Invalid^d, e^ | 4 (4.3) | 8 (5.1) |  | 2 (6.1) | 1 (0.7) |

Abbreviations: iHUU, HIV-unexposed uninfected infants; iHEU, HIV-exposed uninfected infants; SFCs, spot-forming cells; PHA, phytohemagglutinin.

^a^Positive if there were ≥ 8 SFCs above Nil control for at least one of TB antigens.

^b^Negative if a test did not fall into any of the interpretations.

^c^Borderline if the difference to Nil controls was between 5-7 SFCs for at least one of TB antigens.

^d^Invalid if there was PHA < 20 SFCs or Nil control >10 SFCs.

^e^Reason of invalid: PHA < 20 SFCs (n = 6) and Nil control >10 SFCs (n = 6) in South Africa; PHA < 20 SFCs (n = 1) and contamination of kits or assay (n = 2) in Botswana.
